# Erratum: Influence of Composition and Plasma Power on Properties of Film from Biodegradable Polymer Blends. *Polymers* 2020, *12*, 1592

**DOI:** 10.3390/polym12091998

**Published:** 2020-09-02

**Authors:** Leona Omaníková, Ján Bočkaj, Mirko Černák, Roderik Plavec, Jozef Feranc, Patrik Jurkovič

**Affiliations:** 1 Institute of Natural and Synthetic Polymers, Faculty of Chemical and Food Technology, Slovak University of Technology, Radlinského 9, 812 37 Bratislava, Slovakia; jan.bockaj@stuba.sk (J.B.); roderik.plavec@stuba.sk (R.P.); jozef.feranc@stuba.sk (J.F.); patrik.jurkovic@stuba.sk (P.J.); 2 CEPLANT, Department of Physical Electronics, Faculty of Science, Masaryk University, Kotlarska 2, 611 37 Brno, Czech Republic; cernak@physics.muni.cz

The authors wish to make a change to the published paper [1]. We wish to add an affiliation of Leona Omaníková and Ján Bočkaj—CEPLANT, Department of Physical Electronics, Faculty of Science, Masaryk University, Kotlarska 2, 611 37 Brno, Czech Republic. Part of the research was conducted at the CEPLANT center. Mrs. Omaníková and Mr. Bočkaj have a combined affiliation at STU and CEPLANT, as part of a contract between STU and CEPLANT.

The authors apologize for any inconvenience caused and the change does not affect the scientific results. The manuscript will be updated, and the original will remain online on the article webpage at https://www.mdpi.com/2073-4360/12/7/1592.
